# Hepatitis E virus screening in solid organ transplant recipients: prevalence and implications for implementation, Spain, 2021 to 2023

**DOI:** 10.2807/1560-7917.ES.2025.30.44.2500254

**Published:** 2025-11-06

**Authors:** Sara Pereira, Alfredo Pérez-Rivilla, Raquel Carracedo, Pedro López-López, Manuel Rodríguez-Iglesias, Rafael Benito, Ana Fuentes, Miguel Ángel López-Ruz, Carolina Freyre-Carrillo, María Del Valle Odero, Noelia Parajó, Federico García, Antonio Rivero, Antonio Rivero-Juárez, Antonio Aguilera

**Affiliations:** 1 Clinical Microbiology Unit, Hospital Clínico Universitario de Santiago de Compostela, Instituto de Investigación Sanitaria de Santiago (IDIS), Santiago de Compostela, Spain; 2 Clinical Microbiology Unit, Hospital Universitario 12 de Octubre, Madrid, Spain; 3 Clinical Virology and Zoonoses Research Group, Unit of Infectious Diseases, Hospital Universitario Reina Sofía, Instituto Maimónides de Investigación Biomédica de Córdoba (IMIBIC), Universidad de Córdoba (UCO), Córdoba, Spain; 4 Centro de Investigación Biomédica en Red de Enfermedades Infecciosas (CIBERINFEC), Instituto de Salud Carlos III, Madrid, Spain; 5 Clinical Microbiology Unit, Hospital Universitario Puerta del Mar, Instituto de Investigación e Innovación Biomédica de Cádiz (INIBICA), Cádiz, Spain; 6 Clinical Microbiology Unit Hospital Clínico Universitario Lozano Blesa, Zaragoza, Spain; 7 Clinical Microbiology Unit, Hospital Universitario San Cecilio, Instituto de Investigación Biosanitaria Ibs, Granada, Spain; 8 Clinical Microbiology Unit, Hospital Universitario Virgen de las Nieves, Granada, Spain; 9 Clinical Microbiology Unit, Hospital Universitario de Puerto Real, Puerto Real, Spain; 10 Clinical Microbiology Unit, Hospital del S.A.S. de Jerez de la Frontera, Jerez de la Frontera, Spain; 11 Instituto de Investigación del Medio Acuático para una Salud Global (iARCUS), Universidad de Santiago de Compostela (USC), Santiago de Compostela, Spain; * These authors contributed equally to this work and share last authorship.

**Keywords:** Hepatitis E virus, solid organ transplant, molecular screening, chronic infection, Spain

## Abstract

INTRODUCTION: In solid organ transplant (SOT) recipients, hepatitis E virus (HEV) poses a complex clinical challenge, because there is a risk of developing persistent infection. Current European guidelines recommend the screening of HEV in SOT recipients because of the risk of unrecognised infection. Despite these recommendations, routine screening for HEV remains underutilised in clinical practice.

AIM: Our study aimed to determine the prevalence of HEV viraemia and to evaluate the clinical evolution of HEV infection in SOT.

METHODS: We performed a multicentre cross-sectional study including adult SOT recipients under follow-up in Spain. All patients were prospectively tested for HEV RNA in peripheral blood. Individuals with detectable viraemia were prospectively followed up every 3 months to evaluate viral persistence.

RESULTS: A total of 940 patients were included in the study. Five patients were infected, supposing a prevalence of HEV infection in Spanish SOT recipients of 0.53% (95% confidence interval: 0.23–1.24). Genotyping was successfully conducted in four cases, all identified as genotype 3. All patients were asymptomatic and had varied levels of liver enzyme elevations. At follow-up, three of the five patients remained HEC RNA-positive, consistent with chronic infection. Overall, the prevalence of chronic HEV infection in our study population was 0.32%.

CONCLUSION: Our study highlights the need to establish periodic molecular HEV surveillance in SOT recipients.

**Key public health message**

**What did you want to address in this study and why?**

Hepatitis E virus (HEV) infection is an emerging concern for solid organ transplant recipients. It can lead to chronic disease and severe complications because patients need to take medication that suppresses the immune system. Current guidelines recommend annual HEV screening in this population, but the implementation varies across countries as data on the infection’s prevalence and impact are limited.

**What have we learnt from this study?**

We systematically screened solid organ transplant recipients across Spain and found that about one in 200 had hepatitis E virus infection, and three of five infected developed chronic disease. These findings are consistent with previous reports on the and clinical burden of HEV infection in transplant patients.

**What are the implications of your findings for public health?**

Our results support the implementation of a Spanish national HEV screening programme to enable early detection, timely treatment and improved patient outcomes, ultimately reducing healthcare costs associated with prolonged hospitalisations.

## Introduction

Hepatitis E virus (HEV) is responsible for considerable global morbidity [1], ranked as one of the major causes of viral hepatitis worldwide [2]. While HEV genotypes 1 and 2 are predominantly seen in countries with poor sanitation and are primarily transmitted via the faecal-oral route [3], genotypes 3 and 4 occur globally, and are primarily transmitted through consumption of raw or undercooked meat (mainly game and pork) [4]. The clinical presentation of HEV infection in immunocompetent individuals is generally an asymptomatic or mild, self-limiting acute hepatitis [5]. However, in certain populations, including those with pre-existing liver disease or immunocompromised states, HEV infection can cause acute liver failure, particularly in pregnant women [6], or can lead to extrahepatic manifestations with neurological or renal involvement [7].

In the context of immunocompromised patients, particularly solid organ transplant (SOT) recipients, HEV poses a more complex clinical challenge. These patients are at risk of developing persistent or chronic HEV infection [8], which is defined by the persistence of HEV RNA in serum or stool for more than 3 months [9]. Chronic HEV infection is particularly concerning in this population as it can lead to progressive fibrosis and cirrhosis. As a result, identification and management of HEV infection in these patients are critical, and there is growing recognition of the need for systematic screening to detect asymptomatic or chronic cases [10].

Current European guidelines recommend the screening of HEV in patients undergoing immunosuppressive therapy [9-11], particularly SOT recipients, because they are at risk of unrecognised chronic infection and subsequent liver damage. Despite these recommendations, routine screening for HEV remains underutilised in clinical practice outside of a few specific regions. In the United Kingdom (UK), screening has proven to be a cost-effective strategy [12], particularly in regions where HEV prevalence is high, as evidenced by studies showing prevalences of active infection of 0.46% and 0.67% among SOT recipients [13,14]. In contrast, there is a notable gap regarding HEV screening for viraemia and chronic infection in other countries, particularly in southern Europe. Understanding the epidemiology of HEV infection in different settings is crucial for the development of evidence-based guidelines and targeted screening programmes. Thus, our study aimed to assess the prevalence, chronicity and molecular epidemiology of HEV infection in SOT populations in nine reference hospitals of the Spanish Health System, based on systematic screening during routine follow-up.

## Methods

### Study concept and design

The study was coordinated by the Spanish Study Group for Viral Hepatitis (GEHEP) of the Spanish Society of Clinical Microbiology and Infectious Diseases (SEIMC). We included all adult SOT recipients who had a clinical visit scheduled at any of the participating hospitals during the study period (2021–2023). No additional inclusion or exclusion criteria were applied. As part of the visit, HEV RNA testing was incorporated into the routine laboratory evaluation, following current European recommendations [9-11], regardless of liver enzyme levels or clinical suspicion of infection. The participating hospitals were located in four autonomous communities (Andalusia, Galicia, Madrid and Aragon) and across six different provinces (A Coruña, Madrid, Córdoba, Cádiz, Zaragoza and Granada). According to the 2024 National Transplant Registry, Spain has 47 hospitals authorised to perform solid organ transplants. The centres participating in this study represent ca 19% of all hospitals authorised to perform transplants in Spain, covering around 30% of the national transplant activity. Collectively, these centres serve a general population of ca 11.8 million people, representing around 24% of the total Spanish population. This design enabled the systematic screening of the transplant population under active follow-up and allowed for an accurate estimation of HEV viraemia prevalence in a real-life clinical setting, while also assessing the feasibility of implementing systematic screening within existing care pathways.

### Hepatitis E virus marker evaluation and definitions

All patients included in the study were tested for HEV RNA. In line with current recommendations for immunosuppressed patients, serological testing was not used for screening purposes, as this method may yield false-negative results due to delayed or absent antibody responses and is therefore not considered reliable in this setting. Instead, viral RNA detection was performed using either the COBAS HEV test (Roche Diagnostics, Switzerland) or the RealStar HEV RT-PCR Kit (Altona Diagnostics, Germany), depending on availability in the local laboratory. According to the product inserts, the limit of detection (LoD) for the COBAS HEV test is 6 international units (IU)/mL, and for the RealStar HEV RT-PCR Kit, it is 20 IU/mL. Both tests include the use of positive and negative controls, as well as an internal control for RNA extraction to ensure assay validity. 

We defined acute HEV infection as the presence of detectable HEV RNA in serum. Samples with detectable HEV RNA were sequenced at a central reference laboratory (Instituto Maimonides de Investigacion Biomedica de Cordoba (IMIBIC)) using a standardised and harmonised protocol described elsewhere [15]. Briefly, samples were genotyped by targeting the ORF2 region using primers HEV_5920S (5′-CAAGGHTGGCGYTCKGTTGAGAC-3′) and HEV_6425A (5′-CCCTTRTCCTGCTGAGCRTTCTC-3′) in the first round, and primers HEV_5930S (5′-GYTCKGTTGAGACCWCBGGBGT-3′) and HEV_6334A (5′-TTMACWGTRGCTCGCCATTGGC-3′) in the second round. The second amplification product of 467 bp was sequenced using a BigDye Terminator cycle sequencing ready reaction kit on an ABI Prism 3100 genetic analyser (Applied Biosystems, Foster City, CA, US). SnapGene software (Version 3.1; GSL Biotech) was used for sequence analysis. These patients were subsequently followed every 3 months until the end of the study period to evaluate viral persistence. We defined chronic HEV infection as persistent detection of HEV RNA in blood for more than 3 months. In addition, in patients with detectable viral RNA, serological testing for anti-HEV IgG and IgM was performed locally using VirClia Lotus (VIRCELL, Spain).

### Variable collection

In all patients, we systematically collected demographic, clinical and laboratory data. The demographic variables included sex (male or female) and age recorded in years. Clinical data encompassed the type of organ transplanted, categorised as renal, liver, heart, lung, or combined renal and liver transplants. In addition, we documented the immunosuppressive therapy regimen and the major underlying condition necessitating transplantation. We also collected information regarding co-infections with other hepatotropic viruses, specifically hepatitis C virus (HCV) and hepatitis B virus (HBV). Laboratory parameters assessed included alanine aminotransferase (ALT) levels (units (U)/L), aspartate aminotransferase (AST) levels (U/L), γ-glutamyl transferase (GGT) levels (U/L), and total bilirubin levels (mg/dL). Each hospital adheres to rigorous quality control standards, including ISO 15189 for medical laboratories and ISO 9001 for quality management systems. These standards ensure robust procedures for accuracy, precision and reliability in testing, with strict instrumentation controls and internal validation mechanisms in place. Symptoms were evaluated and recorded, including fever, nausea, vomiting, jaundice, acholic stools, choluria, diarrhoea, abdominal pain, arthralgia, fatigue, pruritus and any extrahepatic manifestations.

### Statistical analysis

The primary outcome variable of the study was the presence of HEV RNA in serum. We calculated the overall prevalence of HEV viraemia and the proportion of patients with persistent RNA detection after 3 months, defined as chronic infection [9-11]. Continuous variables were summarised using the median and interquartile range (IQR), and categorical variables are reported as absolute values and percentages. We used two-sided 95% confidence intervals (CI) for proportions via the exact binomial distribution.

Consensus sequences from positive samples were obtained using SeqMan NGen Software (Version 12.0, DNASTAR, Madison, WI, US). We performed HEV genotype assignments using the HEVnet genotyping tool and confirmed using the Basic Local Alignment Search Tool (BLASTn). We generated sequence alignments with the Multiple Alignment using Fast Fourier Transform (MAFFT) online service, and constructed the phylogenetic tree with the maximum likelihood method. We inferred the evolutionary history using the maximum likelihood method based on the Jukes–Cantor model. The bootstrap consensus tree inferred from 1,000 replicates is taken to represent the evolutionary history of the taxa analysed. Initial tree(s) for the heuristic search were obtained automatically by applying neighbour-joining and BioNJ algorithms to a matrix of pairwise distances estimated using the maximum composite likelihood approach; we then selecting the topology with superior log likelihood value.

## Results

### Study population

A total of 940 patients were included in the study, with a predominance of males (64.3%). The median age at study entry was 61 years (IQR: 52–68). Renal transplant recipients represented 60.1% of the cohort (n = 565), followed by liver (31.0%; n = 291), lung (7.7%; n = 72), combined renal and liver (1.1%; n = 10) and heart transplant recipients (0.1%; n = 1). A major underlying condition was identified in 108 patients (11.5%); this included liver cirrhosis in 54 patients (5.7%), solid organ malignancies in 25 (2.6%), autoimmune or inflammatory diseases in 21 (2.2%), HIV infection in six (0.6%) and haematologic malignancies in two (0.2%). All details are presented in Table 1.

**Table 1:** Characteristics of solid organ transplant patients screened for hepatitis E virus infection, Spain, 2021–2023 (n = 940)

Variable	n	%
Sex		
Male	605	64.4
Female	335	35.6
Median age in years (IQR)	61 (52–68)	
Organ transplanted		
Renal	565	60.1
Liver	291	31.0
Lung	72	7.7
Renal + liver	11	1.2
Heart	1	0.1
Major underlying condition	108	11.5
Liver cirrhosis^a^	54	5.7
Malignancy^b^	25	2.7
Autoimmune disease^c^	21	2.2
HIV	6	0.6
Haematological malignancies^d^	2	0.2

IQR: interquartile range.

^a^ Alcoholic cirrhosis (n = 36), hepatitis B virus infection (n = 10), hepatitis C virus infection (n = 8).

^b^ Hepatocellular carcinoma (n = 15), colorectal (n = 5), prostate (n = 2), lung (n = 2), breast cancer (n = 1).

^c^ Inflammatory bowel disease (n = 5), idiopathic pulmonary fibrosis (n = 5), multiple sclerosis (n = 1), psoriasis: (n = 10).

^d^ Lymphoma (n = 1), myeloid malignancy (n = 1).

### Hepatitis E virus screening

A total of five patients showed detectable HEV RNA in serum, corresponding to a prevalence of HEV viraemia in the study population of 0.53% (95% CI: 0.23–1.24). Genotyping was successful in four cases, all viruses were identified as genotype 3, of which three were assigned as 3f1(PQ869629, PQ869630 and PQ869631) and one as 3m (PQ869628). The phylogenetic analysis of the sequences and their relation with sequences found in human and swine from near areas is shown in the Figure.

**Figure:**
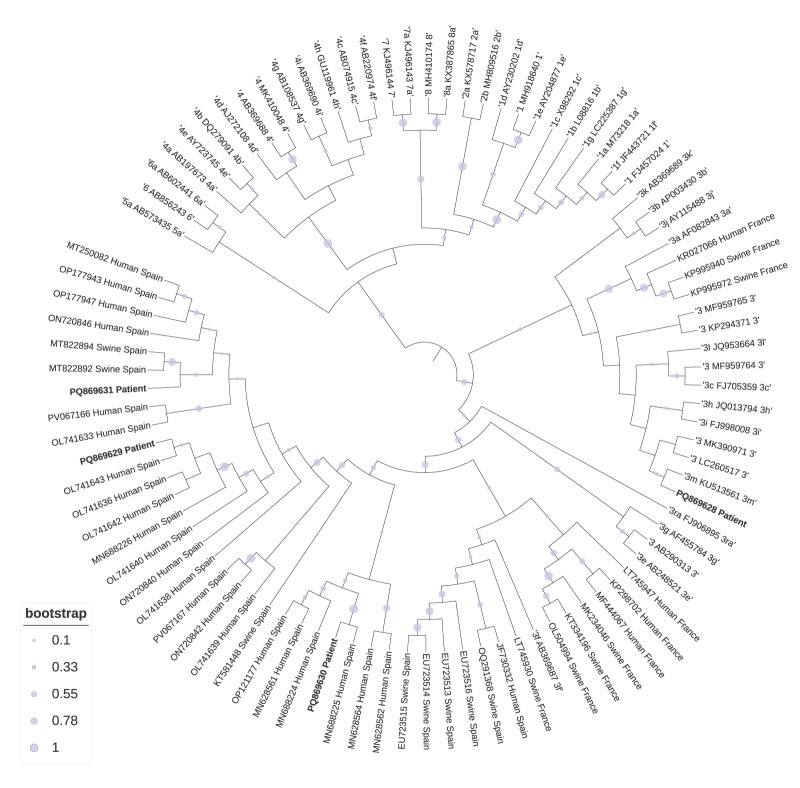
Molecular phylogenetic analysis of hepatitis E virus from solid organ transplant patients, Spain, 2021–2023 (n = 5)

The evolutionary history was inferred by using the maximum likelihood method based on the Jukes–Cantor model. The bootstrap consensus tree inferred from 1,000 replicates is taken to represent the evolutionary history of the taxa analysed. Initial tree(s) for the heuristic search were obtained automatically by applying neighbour-joining and BioNJ algorithms to a matrix of pairwise distances estimated using the maximum composite likelihood approach and then selecting the topology with superior log likelihood value. The analysis involved 93 sequences. Codon positions included were 1st + 2nd + 3rd + noncoding. All positions containing gaps and missing data were eliminated. There were a total of 472 positions in the final dataset. Evolutionary analyses were conducted in MEGA6. Sequences obtained in this study are highlighted in bold.

Table 2 summarises the main clinical and demographic characteristics of patients with detectable HEV RNA. Among these five patients, three were male and two were female, with ages ranging from their mid-30s to mid-60s. All patients were asymptomatic and had varied levels of liver enzyme elevations. Their AST, ALT and GGT levels ranged from mild increases (18/16/16 U/L) to significant elevations (673/1,425/183 U/L). Total bilirubin levels were within normal or slightly elevated ranges (0.7–2.2 mg/dL). The infected individuals included patients with lung, renal and liver transplants. Immunosuppressive therapy varied, with tacrolimus the most commonly used agent, either alone or in combination with other drugs such as sirolimus, everolimus or corticosteroids. All patients tested positive for HEV IgG and IgM antibodies. Three of five patients had detectable HEV RNA 3 months after diagnosis, indicating chronic infection. The overall prevalence of chronic HEV infection in the study population was 0.32% (95% CI: 0.07–0.93).

**Table 2:** Characteristics of solid organ transplant patients with detectable hepatitis E virus RNA, Spain, 2021–2023 (n = 5)

Year of transplant	AST/ALT/GGT (U/L)^a^	Total bilirubin (mg/dL)^b^	Organ	Immunossupressive therapy	HEV RNA (IU/mL)^c^	IgG/IgM	Development of HEV chronic infection	Genotype (GenBank)
2012	18/16/16	2.2	Lung	Tacrolimus	45,000	Pos/pos	Yes	NA
2018	33/27/31	0.7	Renal	MM + Tacrolimus + Corticosteroid	1,659,418	Pos/pos	Yes	3m (PQ869628)
2013	154/225/105	0.9	Renal	Tacrolimus + Sirolimus + Corticosteroid	NQ	Pos/pos	Yes	3f1 (PQ869629)
2019	673/1,425/183	0.7	Renal	Tacrolimus + Everolimus + Corticosteroid	NQ	Pos/pos	No	3f1 (PQ869630)
2010	50/63/811	1.3	Liver	Everolimus + Corticosteroid	NQ	Pos/pos	No	3f1 (PQ869631)

ALT: alanine aminotransferase; AST: aspartate aminotransferase; ID: patient identification number; F: female; GGT: γ-glutamyl transferase; HEV RNA: hepatitis E virus viral load; IgG: immunoglobulin G; IgM: immunoglobulin M; IU: international units; M: male; MM: mycophenolate mofetil; NA: not available; NQ: not quantified; pos: positive; U: units.

^a^ Reference values for liver enzymes: AST (13–40 U/L), ALT (7–40 U/L) and GGT (3–60 U/L).

^b^ Reference range for total bilirubin was 0.2–1.3 mg/dL.

^c^ Viral load was only obtained when using the COBAS HEV test (Roche Diagnostics, Switzerland). When using the RealStar HEV RT-PCR Kit (Altona Diagnostics, Germany), quantification was not possible.

## Discussion

European guidelines recommend annual HEV screening in SOT recipients because of the risk of chronic infection [9-11]. The clinical importance of undiagnosed HEV infection is supported by recent Spanish national data showing that advanced solid organ transplantation is a significant risk factor for prolonged hospitalisation due to hepatitis E [16]. Preventing complications through early detection could reduce not only morbidity but also the economic burden associated with extended hospital stays [17]. Early diagnosis is essential to enable timely liver assessment and targeted management, such as adjusting immunosuppressive therapy or initiating antiviral treatment, both aimed at achieving viral clearance and preventing complications [18]. In our cohort, HEV viraemia was detected in 0.53% of patients, a prevalence consistent with reports from other European countries [13,14,19-22] and the United States (US) [23]. Moreover, three of five viraemic patients developed chronic infection, highlighting the potential long-term consequences of undetected HEV in immunosuppressed individuals. Although assessing feasibility was not a predefined objective, the study was embedded in routine transplant care, incorporating HEV RNA testing into scheduled follow-up visits without altering standard procedures. This pragmatic design allowed us to estimate viraemia prevalence under real-world conditions.

The rate of chronic infection in our study was similar to those observed in other studies of immunosuppressed populations, where more than 60% of solid organ transplant recipients with HEV infection progressed to chronic hepatitis [18]. This resulted in an overall chronic HEV infection prevalence of 0.32% in our cohort, emphasising the risk of persistent infection in transplant recipients under immunosuppressive therapy. Remarkably, all three chronically infected patients were receiving tacrolimus, a calcineurin inhibitor identified as an independent risk factor for chronic HEV infection [18]. While routine screening of at-risk populations could be beneficial, it is unclear whether a selective or nonselective approach would be more effective in identifying persistent HEV infections [12,14]. A selective strategy could focus on populations with known higher viraemia rates or be guided by clinical indicators such as specific immunosuppressive regimens or prior exposure to blood products [14]. However, identifying these patients can be challenging in microbiology laboratories that manage a high workload. A British study evaluated several diagnostic strategies and concluded that annual HEV RNA screening of all patients yielded the highest increase in quality-adjusted life years. However, this approach was limited by the high cost of molecular testing. As a result, screening of patients with ALT levels outside the normal range was a more cost-effective option [12]. Thus, guidelines advise HEV testing in SOT recipients with elevated liver enzymes or symptoms suggestive of HEV infection [9,10,24]. However, we identified patients with normal liver enzyme levels who were infected with HEV, a finding echoed in other studies [9,14,18]. As HEV infections can be asymptomatic or present with mild symptoms, we advocate for a routine HEV screening strategy in transplant recipients that includes RNA testing, regardless of liver enzyme levels, to ensure that infections are not overlooked.

According to current guidelines, HEV nucleic acid testing (NAT) is the recommended strategy for screening immunosuppressed patients, including solid organ transplant recipients [9]. However, NAT is costly and may not be universally accessible. A cost-effectiveness analysis conducted in the UK found that universal NAT screening was cost-effective in ca 80% of simulated scenarios, particularly when HEV prevalence exceeded 0.4% [12]. Furthermore, the observed prevalence of HEV viraemia in our cohort exceeded the threshold established as cost-effective in previously published models [12]. While formal cost-effectiveness analyses are needed in the Spanish context, these results suggest that systematic HEV screening in transplant recipients may be both clinically valuable and economically reasonable. Nevertheless, the same study indicated that more economical alternatives could offer favourable cost-effectiveness under certain conditions. These limitations underline the need to evaluate alternative, more accessible screening strategies. Anti-HEV IgM, a classical marker of acute infection, is generally not recommended in this population because seroconversion may be delayed or absent in immunosuppressed patients [9]. Interestingly, in our study, all patients with detectable HEV RNA also tested positive for anti-HEV IgM. While this finding suggests that IgM detection might be useful in some transplant recipients, our study did not account for the time since transplantation, and it is therefore possible that antibody production may vary depending on immune status and time post-transplant. On the other hand, HEV antigen detection could represent a feasible alternative [9]. In the UK cost-effectiveness model, antigen-based screening demonstrated good performance in specific contexts, Overall, that study supports the implementation of targeted HEV-Ag screening in transplant recipients with abnormal liver function tests as a highly cost-effective and potentially cost-saving public health intervention. However, in our study three of five patients with detectable HEV RNA presented with normal ALT levels, therefore a screening strategy based on ALT elevation would have failed to detect these cases. Moreover, although HEV antigen testing may be more affordable, its sensitivity is lower than that of RNA detection, and it is not currently recommended as a first-line screening tool in immunocompromised individuals [9,10,24]. 

This study has several limitations. Firstly, the number of patients with detectable HEV RNA was low, which limits the generalisability of the findings and precludes any analysis of risk factors, clinical progression or genotype-specific outcomes [25]. Secondly, although the cross-sectional design allowed us to estimate the prevalence of active HEV infection in this population, it does not permit assessment of incidence. Since incidence is the key metric for evaluating the potential effectiveness and impact of screening interventions over time, this is an important limitation when considering implementation of a systematic screening strategy. Thirdly, while our results may support future policy decisions, we did not perform a formal cost-effectiveness analysis, which would be essential to justify the economic viability of national screening programmes. Fourthly, HEV antigen testing was not performed, so its performance could not be compared with RNA detection. Fifthly, the study did not include individual-level data on potential HEV exposure risks, such as dietary habits or contact with swine or pork products. While we cannot make direct recommendations, it seems reasonable to minimise the risk of transmission during the critical post-transplant period when immunosuppressive therapy is most intense. These factors may influence infection risk and should be explored in future research. Sixthly, one positive sample could not be successfully sequenced. This is consistent with previous findings evaluating HEV genotyping methodologies, which found that a subset of HEV RNA-positive samples may fail to sequence, even under even under the optimal and harmonised procedure used in our study [26]. Finally, it is important to highlight that while the study included a sample of transplant recipients from several hospitals across different autonomous communities, the results should be considered within the context of the selected centres, which represent ca 19% of the transplant-performing hospitals in the country. Although the sample is diverse, it cannot be claimed to be fully representative of all transplant recipients in Spain. These points highlight the need for larger, prospective studies to identify clinical or virological factors associated with chronic HEV infection, which could help inform more targeted and cost-effective screening strategies in transplant recipients.

## Conclusion

After implementing HEV RNA screening in a representative sample of solid organ transplant recipients in Spain, our study supports the need to establish systematic and annual HEV surveillance using RNA testing, in line with current European guidelines. While HEV RNA remains the most sensitive diagnostic method, future cost-effectiveness analyses should assess the feasibility of alternative approaches such as antigen-based screening.

## Data Availability

All the data generated or analysed during the study are included in this published article. The datasets used and/or analysed during the present research project are available from the corresponding author upon reasonable request. The sequences used in the present study are publicly available in GenBank (PQ869629, PQ869630, PQ869631 and PQ869628).
